# A Bibliometric Analysis of the Literature on Irisin from 2012–2021

**DOI:** 10.3390/ijerph19106153

**Published:** 2022-05-18

**Authors:** Jiangshan Liu, Bote Qi, Lin Gan, Yanli Shen, Yu Zou

**Affiliations:** 1 College of Physical Education, Changzhou University, Changzhou 213164, China; ljs0424913@cczu.edu.cn (J.L.); ganlin@cczu.edu.cn (L.G.); 2 Department of Sport and Exercise Science, College of Education, Zhejiang University, Hangzhou 310058, China; qibote@zju.edu.cn; 3 Library of Shanghai University of Sport, Shanghai 200438, China

**Keywords:** irisin, bibliometric, CiteSpace, VOSviewer, exercise

## Abstract

Irisin is a hormone-like molecule mainly released by skeletal muscles in response to exercise, which is proposed to induce the ‘browning’ of white adipose tissue. Since its identification, irisin was reported to be closely associated with many metabolic diseases, including type 2 diabetes mellitus (T2DM), obesity, cardiovascular disease (CVD), and metabolic bone diseases. In recent years, irisin has attracted increasing research interest, and numerous studies have been published in this field. Thus, it is essential to identify the current research status of irisin and measure research hotspots and possible future trends. In this study, by utilizing two visualization software named CiteSpace and VOSviewer, we analyzed 1510 Web of Science publications on irisin published from 2012 to 2021. Our results show that the number of irisin-related articles published annually has increased significantly. China participates in the most studies, followed by the United States and Turkey. Firat University, Harvard University, and Shandong University are three major institutions with larger numbers of publications. The analysis of keywords co-occurrence indicates that insulin resistance, inflammation, and circulating irisin levels in serum are the research hotspots. Apoptosis, BDNF, and osteoporosis will likely become the focus of future research related to irisin. Overall, this study may provide helpful insights for researchers to understand the current research situation and identify the potential frontiers of irisin.

## 1. Introduction

Irisin, a myokine induced by exercise in mice and humans, is shown to promote energy expenditure by stimulating the ‘browning’ of white adipose tissue [1,2]. It was first discovered in 2012 by Boström et al. and named after the ancient Goddess Iris, the messenger of the Gods [1]. Irisin is secreted from the fibronectin type III domain containing protein 5 (FNDC5) after the cleavage of its extracellular portion. It is mainly released from muscle tissue in response to exercise and exerts beneficial effects on skeletal muscles [3]. In the nervous system, irisin is localized in the paraventricular nucleus of the hypothalamus and the cerebrospinal fluid [4]. A recent study reported that irisin could induce brain-derived neurotrophic factor (BDNF) expression in rat hippocampus and play a key role in the beneficial effects of exercise on synaptic plasticity and memory in Alzheimer’s disease models [5]. Irisin is a glycosylated protein hormone consisting of 112 amino acid residues, and its continuous inter-subunit β-sheet dimer structure (shown in Figure S1) was determined by Schumacher and colleagues [6]. Meanwhile, the receptor for irisin (αV/β5 integrins) was recently identified to display the highest binding ability to irisin by Kim’s group, and they found that irisin functioned through αV/β5 integrins to promote osteocyte survival and sclerostin secretion [7]. In the last decade, numerous studies related to irisin have been conducted, and our understanding of irisin as a potential therapeutic target for various metabolic diseases has made great progress [8,9,10,11]. Until now, irisin remains an interesting molecule from a pathophysiological point of view and is regarded as an exercise mimetic [12]. However, to our knowledge, bibliometric analysis has not been reported in the field of irisin, and the research hot spots and frontiers of irisin remain unclear.

In this study, a bibliometric analysis is conducted to analyze and visualize the research situation and trends concerning irisin. CiteSpace and VOSviewer software are applied in our bibliometric analysis, which could intuitively reveal the dynamic development law of scientific knowledge in a certain period [13,14,15]. Our study may provide a comprehensive understanding of the developments in the research on irisin.

## 2. Materials and Methods

### 2.1. Data Sources and Search Strategies

**Figure 1:**
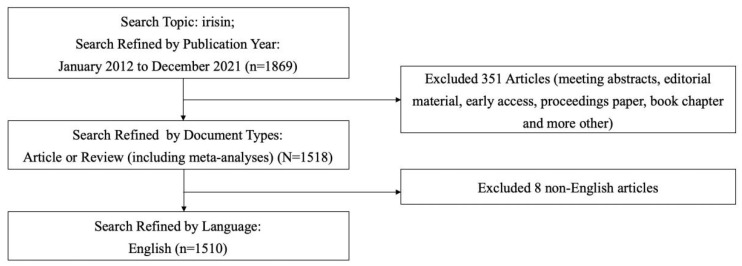
Flow chart of literature screening included in this study.

Literature was retrieved online through the Web of Science Core Collection (WoSCC) on 9 March 2022, with a published time span ranging from 2012 to 2021. The retrieval strategy: Topic words = irisin, Document type = Article and Review (including meta-analyses), Language type = English. This query resulted in 1510 records, which were obtained for this study. We downloaded the original data from WoSCC, selected the full record and cited references, and saved it in plain text format. Each article included data such as topics, authors, abstracts, keywords, titles, publication years, and references. The detailed data retrieval strategies and inclusion criteria for this study are summarized in Figure 1.

### 2.2. Analysis Methods

In this study, Microsoft Excel 2019 (Redmond, Washington, DC, USA) was used to predict the future trend of irisin publications. The equation of the prediction model was as follows: f(x) = ax^3^ + bx^2^ + cx + d, in which x represented the publication year, and f(x) represented the cumulative number of publications. CiteSpace 5.8 R3 software (Drexel University, Philadelphia, PA, USA) was employed to analyze the feature of countries/regions, institutions, co-authorship, journals, and reference co-citation in irisin publications. In addition, VOSviewer 1.6.13 software (Leiden University, Leiden, The Netherlands) was used to analyze the keywords co-occurrence.

## 3. Results

### 3.1. Publication Outputs

**Figure 2:**
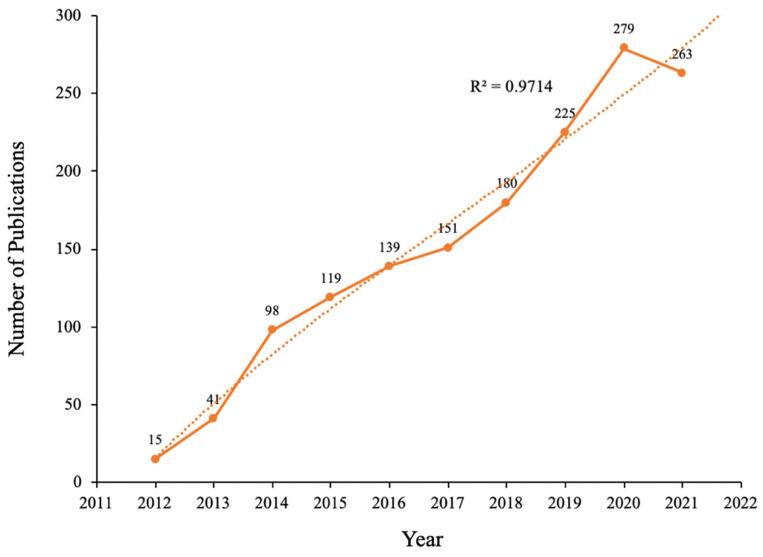
The number of annual publications and growth prediction of irisin research.

The diachronic change in the number of papers, to some extent, reflects the dynamic evolution process of research history and development speed in this field [16]. Figure 2 shows that the number of irisin research papers increased from 15 in 2012 to 263 in 2021. The development growth trend model (R^2^ = 0.9714) predicts that the number of studies being carried out on irisin will increase to 308.

### 3.2. Country Analysis

**Figure 3:**
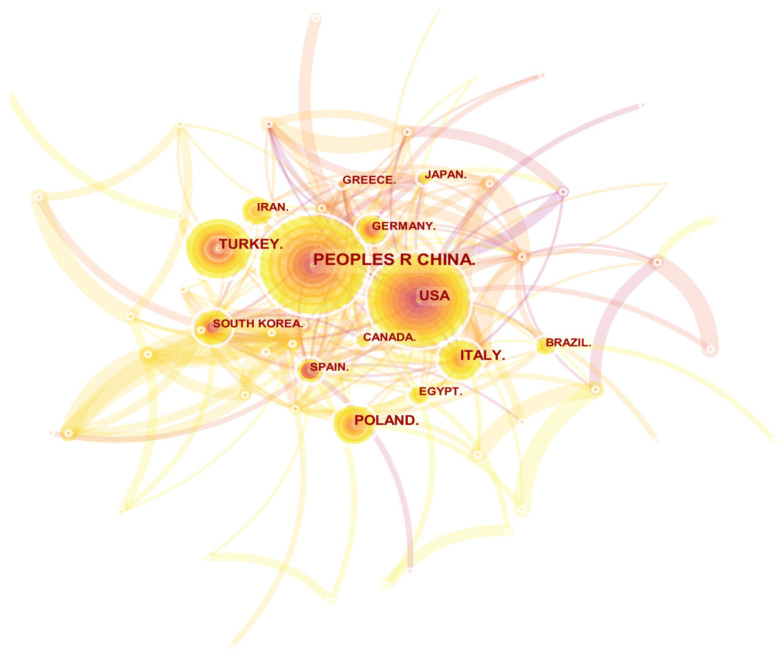
The knowledge mapping of counties/regions in irisin research. Here, each node represents a country, and the size of the node is proportional to the number of publications. The connection represents the cooperative relationship between countries. The thickness of the connection between two nodes represents the intensity of cooperation. The number of connections between nodes represents the degree of cooperation, and the outermost purple of the node represents the centrality. The wider the purple width is, the higher the centrality is, indicating the greater the contribution of the node in the research field.

Through the research on the spatial distribution of a certain field, we can understand the degree of importance a country/region attaches to this research field and the scientific research contributions it has made. The irisin spatial distribution map drawn by CiteSpace is shown in Figure 3. From Figure 3 and Table 1, we can see that China is far ahead of other countries in terms of the number of publications, reaching 376. The United States and Turkey ranked second and third. From the perspective of centrality, the United States, Spain, England, Germany, and Egypt ranked in the top five. The differences between the two rankings show that the research results of European and American countries are more representative and more concerned by researchers. In particular, the United States has published many important kinds of literature with core influence in the field of irisin research.

### 3.3. Institution Analysis

**Figure 4:**
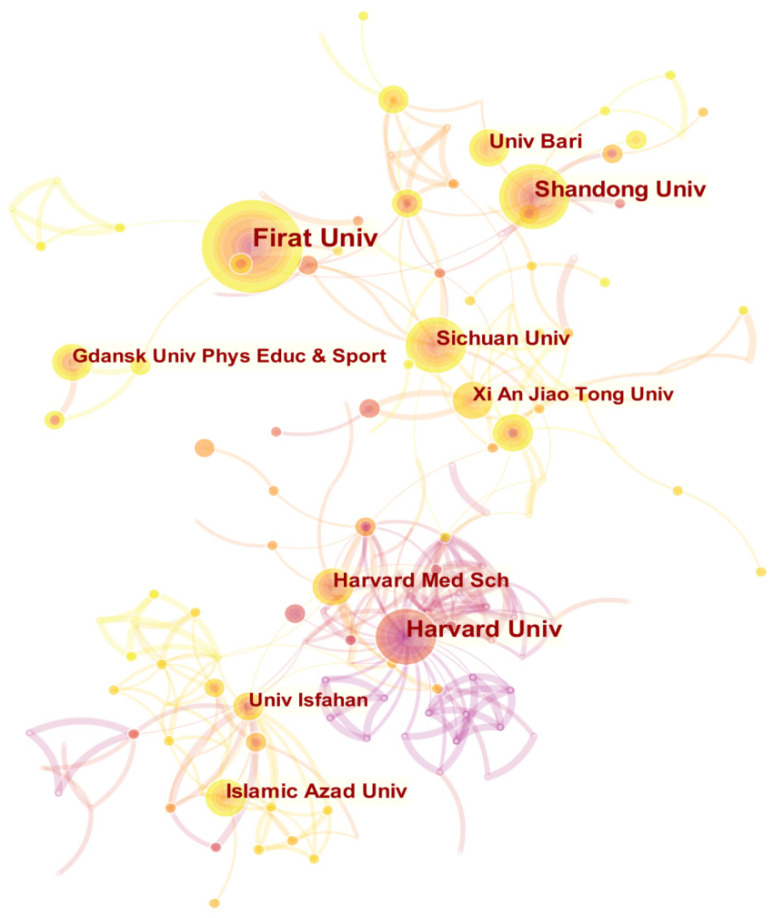
Co-occurrence knowledge map of irisin research institutions. Here, each node represents a scientific research institution, and the size of the node represents the number of published papers of the institution.

In the field of irisin research, the top three research institutions for the number of papers published are Firat University, Harvard University, and Shandong University. From the perspective of centrality, the three research institutions of Sichuan University, Harvard University, and Harvard Medical School are highly central (Table 2). In addition, Figure 4 shows the co-occurrence knowledge map of irisin research institutions drawn by CiteSpace, with a total of 338 nodes. However, there are only 472 connections between nodes, indicating that the research on irisin lacks close cooperation as a whole. The institutions with relatively close cooperation are mainly reflected in the same country. Therefore, more international exchanges and cooperation between different institutions should be strengthened in the future.

### 3.4. Co-Authorship Analysis

**Figure 5:**
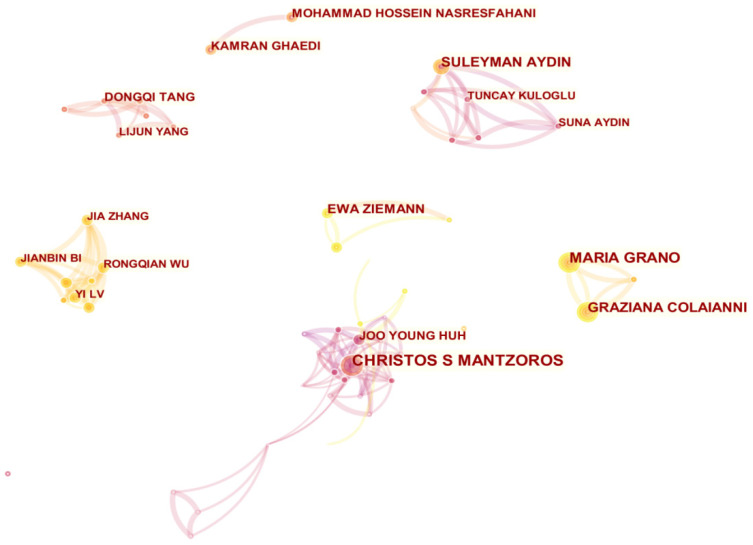
The co-occurrence knowledge map of irisin research authors.

The co-authorship network not only identifies the most productive authors in the field of irisin research but also clearly and visually demonstrates the co-authorship relationship among those authors. We used CiteSpace to visualize the co-authorship network in the field of irisin research. In a co-authorship network map, each node represents an author; the more papers are published, the greater the nodes and fonts are. The connection between nodes indicates the cooperative relationship between authors in this field. As shown in Figure 5, Christos S Mantzoros (25 publications) is identified as the most active author in the field of irisin research, followed by Maria Grano with 19 publications. In addition, this network map has a total of 465 nodes and 1052 connections, and the network density is 0.0098, which indicates that the researchers in this field are not cooperating closely; only a small number of academic teams are formed.

### 3.5. Journal Analysis

According to the statistics of WoSCC, research papers about irisin were published in 393 academic journals. The top 10 journals are presented in Table 3. Among them, the impact factor (IF) and quartile of a journal category are determined according to the 2021 Journal Citation Reports (JCR). *PLoS One* (IF 2020 = 3.240) published the highest number of articles (46 publications, 3.108%), followed by the *International Journal of Molecular Sciences* (34 publications, IF 2020 = 5.924), *Metabolism-Clinical and Experimental* (31 publications, IF 2020 = 8.697), *Scientific Reports* (29 publications, IF 2020 = 4.380), and *Frontiers in Physiology* (27 publications, IF 2020 = 4.566). Among these 10 journals, 4 are published in the United States, and 8 are located in Q1.

### 3.6. Analysis of Keywords Co-Occurrence

**Figure 6:**
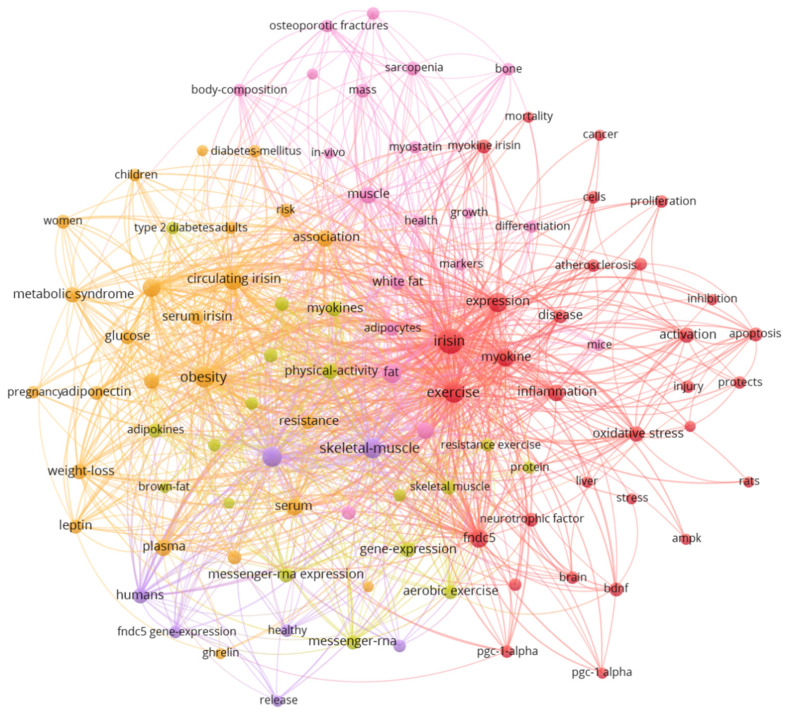
Co-occurrence network of high-frequency keywords in irisin research field.

Keywords facilitate the concentration and refinement of core content and topicality of literature in the research field [17]. The results reveal that the keyword co-occurrence network generated by VOSviewer contains 100 keywords (with a threshold of 25 occurrences) and 3597 co-occurrence links distributed in five clusters (Figure 6). Table 4 summarizes the top 20 high-frequency keywords in irisin’s research. In addition to “irisin”, the top five keywords with the highest frequency are “exercise”, “obesity”, “skeletal muscle”, “adipose tissue”, and “expression”.

### 3.7. Analysis of Reference Co-Citation

**Figure 7:**
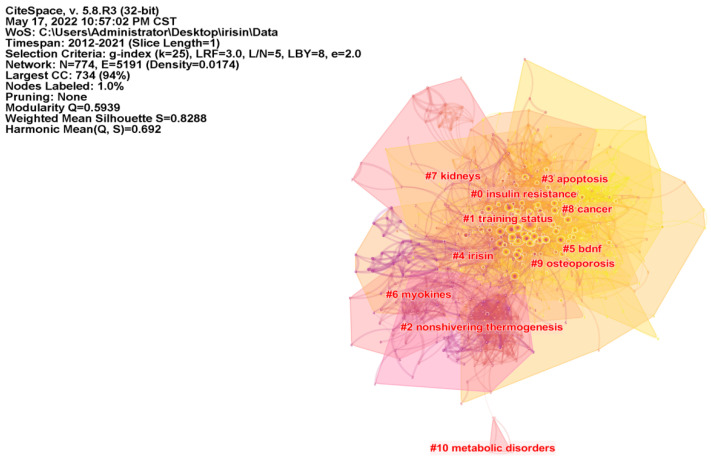
Clusters of reference co-citation network in irisin research. Each node in the figure equals a cited reference, and the larger the node, the higher the citation frequency. In addition, each node in the RCA network can be clustered together according to its inter-connectivity, and each cluster represents a different professional or subject concept.

**Figure 8:**
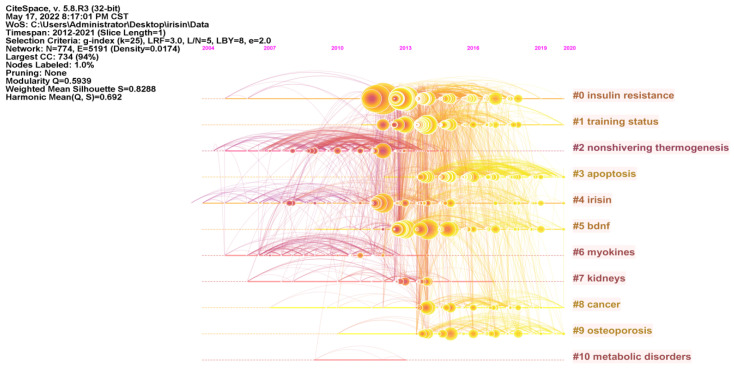
Timeline view of co-cited references related to irisin research.

Reference co-citation analysis (RCA) helps researchers to more intuitively understand the research origin and knowledge base of irisin [13,18]. As illustrated in Figure 7, the network map of RCA consists of the 50 most frequently cited references, with the time slice set as 1 year and the time span set as 2012 to 2021. The visualization of the knowledge network consists of 774 nodes and 5191 links. The modularity Q score of the clustering map is 0.5939, and the weighted mean silhouette value is 0.8288. The largest cluster (Clusters #0) out of 11 clusters is associated with “insulin resistance”, followed by “training status” (Clusters#1), “nonshivering thermogenesis” (Clusters#2), and “apoptosis” (Clusters#3). Furthermore, we analyzed the timeline view for the clusters in Figure 8, “apoptosis” (Clusters #3), “bdnf (brain-derived neurotrophic factor)” (Clusters #5), and “osteoporosis” (Clusters #9) remain active until 2020, indicating that they are the relatively new research directions that recently received widespread attention.

Table 5 shows the top 5 high-frequency co-cited literatures, which lay the foundation for the study of irisin. The literature revealing the origin of irisin displays the highest co-cited frequency. Bostrom et al. pointed out through animal and human experiments that exercise can promote skeletal muscles to secrete a large number of irisin into the blood circulation, promote the browning of white fat, and improve insulin sensitivity and insulin resistance [1]. Thus, irisin may play a role in the prevention and treatment of obesity, type 2 diabetes, and diabetes chronic diseases. The second study conducted by Huh et al. confirmed the expression of irisin in human muscles through an experimental study and suggested that circulating irisin levels could decrease after surgically induced weight loss and increase in response to acute exercise [2]. The third and fourth studies found that irisin could also be secreted from adipose tissue, earning its name as an adipokine. Arturo et al. firstly revealed that white adipose tissue (WAT) also secreted irisin, demonstrating that irisin is not just a myokine liberated by muscle tissue but an adipokine released by adipose tissue [19]. Moreno et al. found that irisin was produced in human preadipocytes and adipocytes and 3T3-L1 adipocytes, and irisin expression in human muscle was 200 times higher than that in adipose tissue; they also concluded that circulating irisin levels were negatively associated with obesity and insulin resistance [20]. The fifth study conducted by Liu et al. firstly reported that circulating irisin is lower in T2DM compared with non-diabetic controls; in the non-diabetic subjects, irisin was positively correlated with several markers of cardiometabolic risks, including fasting glucose, BMI, LDL-cholesterol, total triglycerides, total cholesterol, and diastolic blood pressure [21]. The above five pieces of literature constitute the knowledge basis for the study of irisin, and the centrality of these papers is also high, indicating that they have made important contributions to the research in this field and impacts the subsequent research.

## 4. Discussion

### 4.1. General Information

From the bibliometric analysis of irisin publications over the last 10 years, it was found that the number of articles has increased gradually, reaching a peak in 2020. China has the most published articles, followed by the United States, Turkey, Poland, and Italy. From the perspective of centrality, the United States, Spain, England, Germany, and Egypt rank in the top five. Institutional distribution is generally consistent with country distribution. The top three research institutions for the number of papers published are Firat University, Harvard University, and Shandong University. Christos S Mantzoros (25 publications) is identified as the most active author in the field of irisin research, followed by Maria Grano with 19 publications. However, the researchers in the research field of irisin are not cooperating closely; only a small number of academic teams are formed. The top five co-cited references were published in the years 2012 and 2013, and these pieces of literature lay the foundation for the field of irisin.

### 4.2. Research Hotspots

The analysis of keywords co-occurrence in Figure 6 shows that research of irisin can be divided into five clusters. Based on the frequency and link strength of keywords in the areas, we summarized the following research hotspots of irisin:

#### 4.2.1. Irisin and Insulin Resistance

Insulin resistance is a fundamental pathogenic factor shared by a large number of metabolic disorders [22]. It is inversely correlated to insulin sensitivity and disables their ability to utilize glucose [23]. Irisin was first found to exert browning of WAT and result in body weight loss, emerging as a potential therapeutic target in metabolic diseases [8]. Therefore, research on the relationship between insulin resistance and irisin has always been the focus of researchers in the areas of sports science, medicine, and biology. Bostrom et al. concluded that the increase of circulating irisin levels in mice can effectively increase energy expenditure, reduce body weight, and improve insulin resistance caused by diet [1]. This is the first experiment that reveals that FNDC5/irisin can improve insulin resistance. Moreno et al. then performed in vitro studies in human preadipocytes and adipocytes and induced browning of 3T3-L1 cells and found that circulating irisin levels were negatively associated with obesity and insulin resistance [20]. In human subjects, elevated circulating irisin levels are associated with a lower risk of insulin resistance in obese adults [24,25]; Choi et al. conducted experiments on the normal blood glucose and newly diagnosed T2DM subjects and found that serum irisin levels were decreased in T2DM patients, and the decreased level may be associated with the development of insulin resistance and T2DM, suggesting that irisin played an important role in the pathology of insulin resistance-related disorders [26]. For non-diabetic adults, a previous meta-analysis study identified 15 articles reporting the association between circulating irisin and insulin resistance and concluded that circulating irisin was directly and positively associated with insulin resistance [27]. In addition, irisin could modulate insulin signaling. Yang et al. investigated the effect of irisin on muscle insulin action and found that irisin could promote insulin signaling in myocytes and improve insulin resistance induced by free fatty acids [28]. Zheng et al. reported that irisin improved the PI3K/AKT insulin signaling pathway in both islets of HFD mice and PA-treated MIN6 cells [29]. These studies indicated that irisin played an important role in preserving insulin signaling.

#### 4.2.2. Irisin and Inflammation

Apart from the beneficial effects on insulin resistance, irisin is proposed to possess anti-inflammatory properties. Gannon et al. treated several breast cancer cell lines with the myokine and concluded that irisin might offer therapeutic benefits for breast cancer through an anti-inflammatory response [30]. Zhang et al. evaluated a series of inflammatory markers in T2DM patients and suggested serum irisin would be a new anti-inflammatory factor in protection against T2DM [31]. These studies firstly proposed the potential mechanisms by which irisin exerts anti-inflammatory effects, though the underlying connection between irisin and anti-inflammatory effects has not been studied in detail. The subsequent research suggested that the anti-inflammatory properties of irisin are connected to the suppressed phosphorylation of MAPK and a lower NF-κB activation [32]. Later animal and in vitro studies suggested irisin could exert an anti-inflammatory effect by modulating the expression and activity of cytokines such as TNF-α and IL-6 [33,34]. Nowadays, the anti-inflammatory effects of irisin are reported in various diseases, including but not limited to obesity and type 2 diabetes, cardiovascular diseases, non-alcoholic fatty liver disease, and cancer. Irisin is involved in the anti-inflammatory effects of adipocytes [33], macrophages [32], and β cells [35], activating various signaling pathways. Though the potential anti-inflammatory properties of irisin have been revealed in recent years, further mechanistic studies are still needed to provide additional insights into this research area.

#### 4.2.3. Circulating Irisin Level in Serum

Serum irisin levels can be used as biomarkers for certain diseases. Previous studies have shown that serum irisin concentration in humans alters in different disease conditions [36,37,38,39,40,41,42,43]. In postmenopausal women, the serum irisin level was significantly lower in the sarcopenia group than in the pre-sarcopenia or control groups, and this result suggests that serum irisin may be used as a biomarker for sarcopenia [36]. Another observational cross-sectional study demonstrated that serum irisin concentration was also significantly associated with sarcopenia in patients with liver cirrhosis [37]. Zhang et al. revealed that serum irisin might be a novel biomarker in the diagnosis of hepatocellular carcinoma (HCC), and low preoperative serum irisin levels were significantly correlated with high comprehensive complication index scores after hepatectomy [38]. For the patients with sepsis, chronic kidney disease, and coronary artery disease, serum irisin concentrations were significantly lower in the patients compared with healthy controls and were negatively correlated with disease severity [39,40,41]. However, in a follow-up trial of patients with acute heart failure (AHF), serum irisin was significantly higher in patients deceased in the 1-year follow-up, and its level was associated with mortality [42]. For the patients with hypertension, increased irisin levels were associated with hypertension and hypertension-related stroke [43]. The potential role of serum irisin in the development and progression of diseases would be an interesting area for future investigation. In addition, serum irisin levels could be modulated with exercise intervention. Previous research demonstrated that 8 weeks of high-intensity interval training (HIIT) resulted in beneficial effects in the increase in blood irisin concentration, physical performance, and reduced-fat content [44]. According to Zhao et al., 12-week resistance training intervention significantly increased serum irisin levels in older men, and the reduction in whole-body fat percent was negatively correlated with the increase in serum irisin level [45]. Miyamoto-Mikami et al. showed that an 8-week endurance training program induced an increase in circulating irisin levels, which is associated with a reduction of abdominal visceral fat in middle-aged and older adults [46]. The above results indicated that exercise-training intervention could induce irisin secretion, providing the potential for future therapeutic-related diseases.

### 4.3. Research Frontiers

The timeline view of the knowledge map (Figure 8) reveals that clusters #3 “(apoptosis)”, #5 “(bdnf)”, and #9 “(osteoporosis)” are the focus of current research, highlighting the future development directions of the irisin field.

Apoptosis is a form of programmed cell death. Dysregulation of apoptosis is associated with cancer, neurological disorders, cardiovascular disorders, and autoimmune diseases [47]. Recently, the anti-apoptotic property of irisin has received a great deal of attention from the scientific society. Irisin could promote cell proliferation and inhibit cell apoptosis, including but not limited to pancreatic β cell apoptosis [48], endothelial cell apoptosis [49], and cardiomyocyte apoptosis [50]. Previous studies reported that irisin treatment acts through the Akt [51], ERK [52], p38 MAPK [48], and TLR4/MyD88 [53] signaling pathway to prevent apoptosis and enhance cell survival. A recent study published in 2022 suggested that irisin pretreatment could reduce the apoptosis of adipose tissue-derived mesenchymal stromal cells (ADSCs) and increase the paracrine proangiogenic effect of ADSCs, showing that irisin pretreatment can be an effective means of therapy for ischemic heart injury [54]. For osteocytes, irisin could also increase its function and survival, exerting anti-apoptotic effects [55]. All in all, irisin has been shown to induce anti-apoptotic actions in various diseases, which highlights the therapeutic potential of irisin against diseases.

Brain-derived neurotrophic factor (BDNF) is a key molecule involved in plastic changes related to learning and memory. Exercise can improve cognitive function and is linked to the increased expression of BDNF [56]. However, the underlying mechanisms of exercise-induced BDNF have not been understood yet. A previous study reported that irisin in mice could cross the blood–brain barrier (BBA) and induce hippocampal BDNF through the PGC-1 alpha/FNDC5 pathway [56]. In rats with type 2 diabetes, irisin was shown to regulate the expression of BDNF and glycometabolism and may serve as a promising novel target for the treatment of diabetic mild cognitive impairment [57]. In human subjects, irisin elevation has been known to be associated with the secretion of BDNF [58], and irisin and BDNF levels positively correlated with cognition in the endurance-trained athletes [59]. Furthermore, in patients with Alzheimer’s disease, CSF irisin and BDNF are directly correlated with amyloid-β pathology and cognition [60]. Therefore, irisin/BDNF signaling seems to be an important link between the brain and contracted skeletal muscles during exercise training.

Osteoporosis is a progressive multifactorial skeletal disorder that is characterized by a systemic impairment of bone mass and microarchitecture. It affects millions of individuals (especially postmenopausal women) and remains a clinical challenge in terms of prevention and treatment [61]. As an exercise-induced myokine, irisin serves as a novel biomarker for bone metabolism and has a protective role against osteoporosis [62,63]. Specifically, irisin can increase osteoblasts and decrease the number of osteoclasts, and play a central role in the control of bone mass and quality [64,65]. Qiao et al. studied the signaling pathway by means of which the irisin exerts its osteoblastic effects, and they found that irisin directly targeted osteoblast, promoting osteoblast proliferation and differentiation via activating P38/ERK MAPK signaling cascades [66]. Further experimental studies revealed that recombinant irisin (r-irisin) treatment prevented and ameliorated disuse-induced bone loss in hind-limb suspended mice [67] and improved the mass and strength of cortical bone in healthy mice [65]. In addition, a single-dose administration of irisin to postmenopausal rats with osteoporosis can suppress osteoblast apoptosis and increase trabecular thickness, number, and bone mineral density [68]. In human subjects, a recent meta-analysis study concluded that circulating irisin levels were lower in middle-aged and older adults with osteoporosis, and irisin was positively correlated with bone mineral density [69]. For secondary osteoporosis, cohort studies showed that different pathologies causing secondary osteoporosis (such as primary hyperparathyroidism) are associated with lower circulating levels of irisin [70,71,72]. Thus, irisin may be used as a potential monitoring marker and a valuable treatment target in osteoporosis.

## 5. Conclusions

To our knowledge, this is the first comprehensive quantitative and qualitative bibliometric analysis of scientific documents in the field of irisin research. This study utilizes CiteSpace and VOSviewer software to draw visualization maps, including collaboration among various countries, institutions, and authors, as well as the contribution of journals. The results show that the United States and China have more in-depth research in this field, while international collaboration is still limited. Therefore, it is necessary to further strengthen the cooperation between authors and institutions in various countries to facilitate the sharing of innovative research results. Keywords co-occurrence and reference co-citation analyses provide a reliable perspective for identifying current research hotspots and frontier issues. We found that insulin resistance, inflammation, and circulating irisin levels in serum are the current research hotspots. Apoptosis, BDNF, and osteoporosis will likely become the focus of future research related to irisin. Through further exploration of the irisin, it is helpful to deeply understand the value of irisin and provide new ideas for the treatment of related clinical conditions.

## 6. Limitations

The present study had a few limitations. First, although WoSCC was considered a reliable database source for conducting bibliometric analysis, the data in the present study might not be comprehensive without adding other database resources (e.g., Embase, Medline, and Scopus). Second, we only selected articles published in English, thereby resulting in language bias.

## Figures and Tables

**Table 1:** Spatial distribution information of irisin research.

Rank	Country	Number of Publications	Rank	Country	Centrality
1	Peoples R China	376	1	United States	0.47
2	United States	232	2	Spain	0.26
3	Turkey	156	3	England	0.14
4	Poland	102	4	Germany	0.10
5	Italy	101	5	Egypt	0.10
6	Iran	85	6	Australia	0.09
7	South Korea	79	7	Saudi Arabia	0.09
8	Germany	71	8	Italy	0.07
9	Spain	68	9	Turkey	0.06
10	Brazil	54	10	France	0.06

**Table 2:** High yield institution information of irisin research.

Rank	Institute	Country/Region	Number of Publications	Centrality
1	Firat University	Turkey	54	0.01
2	Harvard University	United States	34	0.12
3	Shandong University	China	32	0.04
4	Sichuan University	China	20	0.14
5	University of Bari Aldo Moro	Italy	19	0.00
6	Islamic Azad University	Iran	19	0.02
7	Harvard Medical School	United States	19	0.09
8	Gdansk University of Physical Education and Sport	Poland	18	0.00
9	Xi An Jiao Tong University	China	18	0.03
10	University Isfahan	Iran	18	0.05

**Table 3:** Ranking of top 10 active journals that published articles on irisin.

Ranking	Journal	Country	Count	IF (2020)	JCR (2020)
1	*PLoS ONE*	United States	46	3.240	Q2
2	*International Journal of Molecular Sciences*	United States	34	5.924	Q1/Q2
3	*Metabolism-Clinical and Experimental*	Netherlands	31	8.697	Q1
4	*Scientific Reports*	England	29	4.380	Q1
5	*Frontiers in Physiology*	Switzerland	27	4.566	Q1
6	*Peptides*	United States	23	3.750	Q2/Q3
7	*Journal of Clinical Endocrinology and Metabolism*	United States	22	5.799	Q1
9	*Frontiers in Endocrinology*	Switzerland	17	5.555	Q1
10	*Nutrients*	Switzerland	15	5.719	Q1

**Table 4:** Top 20 representative keywords in terms of occurrences.

Ranking	Keywords	Occurrences	Total Link Strength	Ranking	Keywords	Occurrences	Total Link Strength
1	irisin	970	5364	11	fndc5	213	1439
2	exercise	491	3261	12	serum	171	1179
3	obesity	412	2791	13	muscle	169	1107
4	skeletal muscle	361	2402	14	association	163	1113
5	adipose tissue	301	2098	15	metabolism	140	902
6	expression	298	1838	16	inflammation	140	801
7	fat	292	1714	17	plasma	129	895
8	circulating irisin	258	1813	18	physical activity	115	763
9	myokine	248	1556	19	humans	110	829
10	insulin resistance	236	1578	20	weight loss	110	785

**Table 5:** High-frequency co-cited literature information of irisin research.

Ranking	Frequency	Author	Title	Year	Journal	References
1	937	Boström, P.	A PGC1α a-dependent myokine that drives the browning of white fat and thermogenesis	2012	*Nature*	[1]
2	472	Huh, J.Y.	FNDC5 and irisin in humans: I. Predictors of circulating concentrations in serum and plasma and II mRNA expression and circulating concentrations in response to weight loss and exercise	2012	*Metabolism-clinical and Experimental*	[2]
3	422	Moreno-Navarrete, J.M.	Irisin is expressed and produced by Human muscle and adipose tissue in association with obesity and insulin resistance	2013	*Journal of Clinical Endocrinology and Metabolism*	[20]
4	314	Roca-Rivada, A.	FNDC5/Irisin is not only a myokine but also an adipokine	2013	*PLoS ONE*	[19]
5	272	Liu, J.J.	Lower circulating irisin is associated with type 2 diabetes mellitus.	2013	*Journal of Diabetes and its Complications*	[21]

## Data Availability

Not applicable.

## Supplementary Materials

The following supporting information can be downloaded at: https://www.mdpi.com/article/10.3390/ijerph19106153/s1. Figure S1: The structures of irisin dimer and one of the irisin receptor (αV/β3).
