# Supplementary material for: A Bibliometric Analysis of the Literature on Irisin from 2012–2021
Source: Int J Environ Res Public Health. 2022 May 18;19(10):6153. doi: 10.3390/ijerph19106153 (PMC9141152; doi:10.3390/ijerph19106153)
Supplement: Supplementary file 1 [file ijerph-19-06153-s001.zip › ijerph-1691032-supplementary.pdf]

International Journal of
Environmental Research and Public Health

MDPI

Article

A Bibliometric Analysis of the Literature on Irisin from 2012–2021

Jiangshan Liu [1], Bote Qi [2], Lin Gan [1], Yanli Shen [3,*], Yu Zou [2,*]

1 College of Physical Education, Changzhou University, Changzhou 213164, China; ljs0424913@cczu.edu.cn (J.L.); ganlin@cczu.edu.cn (L.G.)
2 Department of Sport and Exercise Science, College of Education, Zhejiang University, Hangzhou 310058, China; qibote@zju.edu.cn (B.Q.); zouyuzy@zju.edu.cn (Y.Z.)
3 Library of Shanghai University of Sport, Shanghai 200438, China; shenyanli@sus.edu.cn (Y.S.)
* Correspondence: shenyanli@sus.edu.cn (Y.S.); zouyuzy@zju.edu.cn (Y.Z.); Tel.: +86-021-65506371 (Y.S.); +86-0571-88273934 (Y.Z.)

This material contains one supplemental figure.

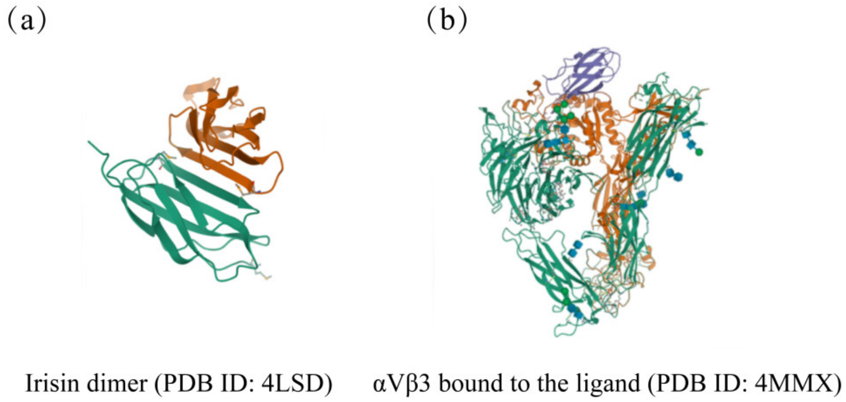

Figure S1. The structures of irisin dimer and one of the irisin receptor (αV/β3).

Citation: Liu, J.; Qi, B.; Gan, L.; Shen, Y.; Zou, Y. A Bibliometric Analysis of the Literature on Irisin from 2012–2021. *Int. J. Environ. Res. Public Health* **2022**, *19*, 6153. https://doi.org/10.3390/ijerph19106153

Academic Editors: Anna Prats-Puig and Sergi Garcia-Retortillo

Received: 5 April 2022
Accepted: 15 May 2022
Published: 18 May 2022

Publisher's Note: MDPI stays neutral with regard to jurisdictional claims in published maps and institutional affiliations.

Copyright: © 2022 by the authors. Licensee MDPI, Basel, Switzerland. This article is an open access article distributed under the terms and conditions of the Creative Commons Attribution (CC BY) license (https://creativecommons.org/licenses/by/4.0/).
